# miR-451 Is a Driver of Lipotoxic Injury in Patients with Diabetic Cardiomyopathy

**DOI:** 10.3390/cells14171401

**Published:** 2025-09-08

**Authors:** Sarah Costantino, Shafeeq A. Mohammed, Federico Ranocchi, Francesco Zito, Valentina Delfine, Nazha Hamdani, Maria Cristina Vinci, Giovanni Melina, Francesco Paneni

**Affiliations:** 1 Center for Translational and Experimental Cardiology (CTEC), Department of Cardiology, University Hospital Zurich and University of Zürich, 8952 Schlieren, Switzerland; 2 Department of Cardiac Surgery, Sant’Andrea Hospital, “Sapienza” University, Via di Grottarossa, 1035 Rome, Italyf.zito@uniroma1.it (F.Z.); giovanni.melina@uniroma1.it (G.M.); 3 Institute of Physiology, Ruhr University, 44801 Bochum, Germany; 4 Department of Molecular and Experimental Cardiology, Ruhr University, 44789 Bochum, Germany; 5 Department of Cardiology, St-Josef Hospital, Ruhr University, 44791 Bochum, Germany; 6 HCEMM-SU Cardiovascular Comorbidities Research Group, Center for Pharmacology and Drug Research & Development, Department of Pharmacology and Pharmacotherapy, Intézet címe Semmelweis University, 1089 Budapest, Hungary; 7 Department of Physiology, University Maastricht, 6211 LK Maastricht, The Netherlands; 8 Centro Cardiologico Monzino IRCCS, Via C. Parea 4, 20138 Milan, Italy

**Keywords:** diabetes, microRNA, lipotoxicity, cardiometabolic disease, epigenetics, HFpEF

## Abstract

MicroRNA 451 (miR-451) is emerging as a pivotal mediator of cardiac damage in experimental models of diabetic cardiomyopathy. Whether miR-451 plays a detrimental role in the human diabetic myocardium is unknown. The present study investigates miR-451’s role in patients with type 2 diabetes (T2D). We show that miR-451 is upregulated in myocardial specimens from T2D patients compared to controls without diabetes and correlates with cardiometabolic parameters, the myocardial triglyceride content and cardiac expression of lipotoxic genes as well as echocardiographic indices of left ventricular dysfunction. Calcium-binding protein 39 (Cab39)—a known target of miR-451 in mouse hearts—was downregulated in T2D patients vs. controls, and its expression negatively correlated with that of miR-451. In cultured human cardiomyocytes (CMs), Ago2 immunoprecipitation confirmed Cab39 to be a direct target of miR-451. Treatment with a high amount of glucose (25mM) and palmitic acid (PA) mimicked miR-451 upregulation and Cab39 downregulation in human CMs. These changes were associated with increased TGs and markers of lipotoxic injury, such as elevated oxidative stress levels, mitochondrial dysfunction and apoptosis. Targeting miR-451 led to restoration of Cab39 levels while rescuing diabetes-induced lipotoxic injury and metabolic dysfunction. By contrast, miR-451 overexpression recapitulated features of lipotoxic damage. Our findings indicate miR-451 to be a potential target for the prevention of myocardial lipotoxic injury in diabetes.

## 1. Introduction

The prevalence of type 2 diabetes (T2D) is skyrocketing across the globe, with 783 million people expected to have the disease by the year 2045 [1]. T2D exerts many deleterious effects on the heart through a combination of hyperglycemic stress and impaired insulin signaling [2,3]. The combined effects of hyperglycemia and insulin resistance impair cardiomyocyte metabolism, fostering lipid accumulation and aberrant fatty acid oxidation with subsequent generation of reactive oxygen species and mitochondrial insufficiency [4]. The mechanisms underlying lipotoxic injury have been extensively studied in experimental models of diabetes and obesity [2,5]. Given the emerging link between metabolic stress and epigenetic changes, experimental work over the last ten years has focused on the role of microRNAs as biological signatures potentially implicated in diabetes-related cardiac damage [6,7,8]. MicroRNAs—defined as small endogenous RNA molecules regulating gene expression at the post-transcriptional level—have emerged as attractive therapeutic targets in the treatment of a wide spectrum of diseases, including heart failure [9]. In cardiac pathology, deregulation of microRNAs’ expression and function is associated with adverse outcomes and heart failure progression [10,11].

Unbiased microRNA profiling in hearts from T2D mice vs. lean controls revealed miR-451 to be a top-ranking microRNA [12]. In mice, this miR was associated with lipotoxic damage and cardiac impairment, whereas its deletion in cardiomyocytes prevented diabetes-induced cardiac hypertrophy and dysfunction [12]. In the aforementioned study, the detrimental action of miR-451 was explained by suppression of AMP-activated protein kinase (AMPK) and upregulation of mammalian target of rapamycin (mTOR) signaling [12]. Although this work provided key insights into the function of miR-451 in experimental models of T2D, its role in the human diabetic myocardium remains unexplored. This aspect deserves attention given the growing impact of miRNA-targeting approaches in patients with cardiomyopathies and heart failure [13]. A first-in-human phase 1b randomized, double-blind, placebo-controlled study recently showed that miR-132 inhibition is safe and is associated with promising beneficial effects in patients with chronic heart failure (HF) [14].

Based on this background, this study was performed to validate this mechanism of myocardial damage in the human diabetic myocardium. Having information on the role of miR-451 in the human heart would pave the way for the development of RNA-targeting approaches to modulate miR-451 and prevent lipotoxic injury in patients with diabetes. Given the emerging role of RNA-targeting therapies [13], the present work aimed at translating an established molecular target identified in mouse models of diabetes into humans.

## 2. Material and Methods

### 2.1. Study Population

T2D patients and age-matched controls without diabetes were consecutively recruited in the Department of Cardiac Surgery, Sant’Andrea Hospital, Rome, Italy. The patients and controls were selected from among those undergoing a cardiopulmonary bypass for surgical valve replacement or coronary artery bypass grafting (CABG). Patients with the following characteristics were excluded: (i) overt signs of cardiomyopathy (i.e., clinical signs of heart failure, a left ventricular ejection fraction of <50%, left atrial dilatation of >40 mm, (ii) a systolic pulmonary artery pressure of >40 mm Hg or a brain natriuretic peptide level of >100 ng/L); (iii) a history of atrial fibrillation/atrial flutter; (iv) stenosis of >50% of the right coronary artery. Patients were also excluded when the amount of tissue was too small for the assessment of its lipid content and molecular analyses. A diagnosis of T2D was made according to the ESC Guidelines [15]. Clinical and echocardiographic data (including from Tissue Doppler Imaging) were obtained at the time of admission. Right atrial tissue was collected during cannulation of the right atrium in preparation for the cardiopulmonary bypass. Atrial samples were immediately prepared for molecular studies, and the remaining tissue was frozen in liquid nitrogen. The study protocol was approved by the Local Ethics Committee and, in accordance with institutional guidelines, all the participants were aware of the investigational nature of the study and gave written consent for their participation.

### 2.2. miR-451 Targeting in Human Cardiomyocytes

In order to mimic the features of T2D in vitro, human cardiomyocytes (AC16 line, Thermo Fisher Scientific, Basel, Switzerland) were cultured in Dulbecco’s modified Eagle’s medium (DMEM) [10% (*v*/*v*) fetal bovine serum (FBS), 100  U/mL penicillin and 100 μg/mL streptomycin at 37 °C in an atmosphere of 95% air and 5% CO_2_] and exposed to palmitic acid (200 μmol/L) and high glucose concentrations (25 mM) for 48 h. Palmitic acid (Sigma, Kanagawa, Japan, P5585) was dissolved in ethanol, and a 500 mmol/L stock solution was stored at −20 °C. This stock solution was combined with 5 mmol/L of fatty acid-free bovine serum albumin (BSA) at a molecular ratio of 10:1 (fatty acid–albumin) in a serum-free medium. In vitro targeting of miR-451 was performed by transfecting cells with 20 nM miR-451 antagomiR or an allStars negative control (Qiagen, Steinhausen, Switzerland).

### 2.3. Real-Time PCR

Total RNA was extracted from human myocardial specimens as well as from human cardiomyocytes using TRIzol Reagent (Invitrogen, Carlsbad, CA, USA) according to the manufacturer’s recommendations. Before extraction, the human myocardial samples were lysed by using a Precellys homogenizer. Conversion of the total cellular RNA to cDNA was carried out with Moloney murine leukemia virus reverse transcriptase and random hexamers (Amersham Bioscience, Piscataway, NJ, USA) in a final volume of 33 μL, using 1 μg of cDNA according to the manufacturer’s recommendations. A real-time PCR was performed using the SYBR Select Master Mix (Applied biosystems, Thermo Fischer Scientific, Zug, Switzerland) on Quant Studio 5 and 7 cyclers (Life Technologies, Thermo Fischer Scientific, Zug, Switzerland) according to the manufacturer’s instructions. GAPDH or TBP was used as an endogenous control for normalizing the RNA concentration. The amplification program consisted of 1 cycle at 95 °C for 10 min, followed by 40 cycles with a denaturing phase at 95 °C for 30 s and a 1 min annealing and elongation phase at 60 °C. A melting curve analysis was performed after amplification to verify the accuracy of the amplicon. The differences in the Ct values between test gene and endogenous controls (GAPDH and TBP, ΔCt) were calculated and used for statistical analysis. All the primers used in this study are reported in Table S1 (Supplementary Materials).

### 2.4. Expression and In Silico Analysis of MicroRNAs

Total RNA isolation was performed using miRNeasy kits from Qiagen and a real-time PCR conducted with miScript Primer Assay (Qiagen) on an MX3000P PCR cycler (Stratagene). The expression of miR-451 was normalized to snord61, according to the manufacturer’s recommendation. In silico prediction analysis was performed by using two different software programs (http://www.targetscan.org/, https://mirdb.org/).

### 2.5. RISC(ago2) Immunoprecipitation

The association of Cab39 mRNA with Ago2 was assayed using a real-time PCR of Cab39 mRNA in Ago2 immunoprecipitates. RISC(ago2) immunoprecipitation was performed using the Magna RIP™ RNA-Binding Protein Kit (Millipore, Aubonne, Switzerland), following the manufacturer’s instructions [16]. Human cardiomyocytes treated with an miR-451 mimic and allStars negative control were lysed in a complete RIP lysis buffer. The supernatants of the cell lysates were incubated with protein G agarose beads for 1 h at 4 °C, after which the precleared samples were incubated with antiAgo2 antibodies (Abcam, Cambridge, UK) or normal mouse IgG-coated protein G agarose beads and rotated overnight at 4 °C. Subsequently, the immune complexes were washed four times with an RIP wash buffer. The total RNAs were isolated using the RNA isolation reagents provided with the kit, followed by a real-time PCR to measure the effects of miR-451 on the interaction of Cab39 mRNA with Ago2.

### 2.6. Triacylglycerol Content in Heart Tissue

The total triglyceride content was determined in human myocardial homogenates using a commercially available kit (Triglyceride Colorimetric Assay Kit, N. 10010303, Cayman Chemicals, Ann Arbor, MI, USA), following the manufacturer’s instructions.

### 2.7. Detection of Lipid Oxidation

Oxidation of lipids, a well-established marker of oxidative stress and lipotoxic injury, was assessed in myocardial specimens from T2D patients and controls by using a lipid peroxidation (MDA) assay kit (Abcam, ab118970), following the manufacturer’s recommendations.

### 2.8. MitoBiogenesis Assay

Mitochondrial biogenesis was assessed by detecting the levels of two mitochondrial proteins (COX1/SDHA) using the in-Cell ELISA Colorimetric kit (Abcam), according to the manufacturer’s instructions.

### 2.9. Mitochondrial Swelling Assay

A total of 40 µg of mitochondria isolated from CMs in a swelling buffer (250 mmol/L sucrose, 10 mmol/L MOPS, 5 µmol/L EGTA, 2 mmol/L MgCl_2_, 5 mmol/L KH_2_PO_4_, 5 mmol/L pyruvate, 5 mmol/L malate) was incubated with 150 µmol/L of calcium chloride (CaCl_2_) in a final volume of 200 µL in a 96-well plate for 20 min, as previously reported [17]. The absorbance was read every 30 s at 520 nm.

### 2.10. Caspase-3 Activity Assay

The Caspase-3 activity was assessed by using a colorimetric activity assay (ab39401, Abcam), according to the manufacturer’s protocol.

### 2.11. Assessment of ATP Content and Fatty Acid Oxidation

The ATP production and fatty acid oxidation were assessed by using commercially available kits (Abcam, Switzerland), according to the manufacturer’s protocol.

### 2.12. Statistical Analysis

Comparisons of continuous variables were performed using an unpaired two-sample *t* test or Mann–Whitney *U* test, as appropriate. Categorical variables were compared using the χ^2^ test. Multiple comparisons were performed using a one-way analysis of variance, followed by Bonferroni correction. Adjusted *p*-values were calculated by multiplying the raw *p*-values by g, where g indicates the number of comparisons. The between-variable correlations were assessed using Spearman’s test. Probability values < 0.05 were considered statistically significant. All the analyses were performed with GraphPad Prism Software (version 7.03).

## 3. Results

### 3.1. miR-451 Is Upregulated in the Human Diabetic Myocardium and Positively Correlates with Lipid Accumulation and Genes Involved in Lipotoxic Injury

**Figure 1:**
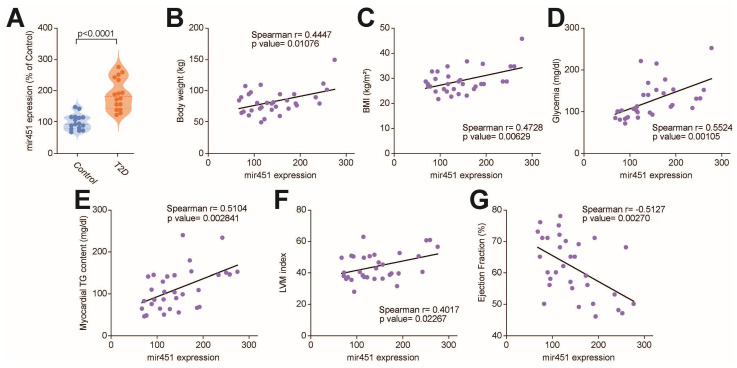
Upregulation of miR-451 in myocardial specimens from patients with type 2 diabetes. (**A**) Real-time PCR shows expression of miR-451 in right atrial specimens from T2D patients compared to subjects without diabetes. Results are presented as mean ± SD and were compared using Student’s *t* test. *p*-value < 0.05 was considered significant. Adjusted *p*-values (alpha/g) are shown. (**B**–**G**) Correlation of miR-451 expression with body weight, body mass index, fasting glucose, myocardial triglyceride content, left ventricular mass and ejection fraction. r indicates Spearman correlation coefficient. BMI, body mass index; EF, ejection fraction; LV mass, left ventricular mass; TG, triglyceride.

**Figure 2:**
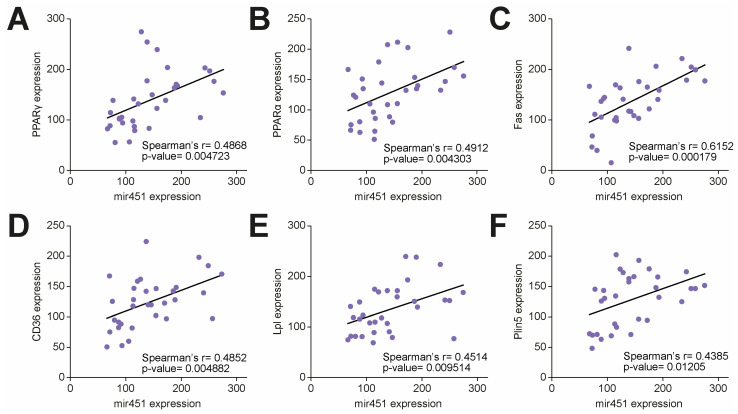
Association of cardiac miR-451 with lipid-related transcriptional programs. (**A**–**F**) Correlation of miR-451 expression with lipid-related transcripts, including PPARγ-, PPARα- and PPAR-dependent genes Fas, Cd36, Lpl and Plin5. r indicates Spearman correlation coefficient. *Fas*, fatty acid synthase; *Lpl*, lipoprotein lipase; *Plin5*, perilipin 5.

Given its pivotal role in experimental models of diabetic cardiomyopathy [12], we first investigated the expression of miR-451 in right atrial specimens collected from T2D patients and age-matched individuals without diabetes undergoing a cardiopulmonary bypass for coronary artery bypass grafting and surgical valve replacement. The T2D patients had a higher BMI and higher fasting plasma glucose and triglyceride levels compared to the subjects without diabetes (Table 1). We found that miR-451 was significantly upregulated in the myocardium of the T2D patients compared to the controls without diabetes (Figure 1A). Cardiac expression of miR-451 positively correlated with cardiometabolic features (body weight, BMI, fasting glucose), the myocardial TG content and the LV mass index, while a negative correlation was observed with the ejection fraction (Figure 1B–G). We next tested the correlation of miR-451 with molecular markers of lipid overload and lipotoxic injury. In the T2D patients, increased cardiac miR-451 levels were positively correlated with gene expression of the PPARγ-, PPARα- and PPAR-dependent genes Fas, Cd36, Lpl and Plin5 (Figure 2A–F).

### 3.2. miR-451 Orchestrates Cab39 Expression

**Figure 3:**
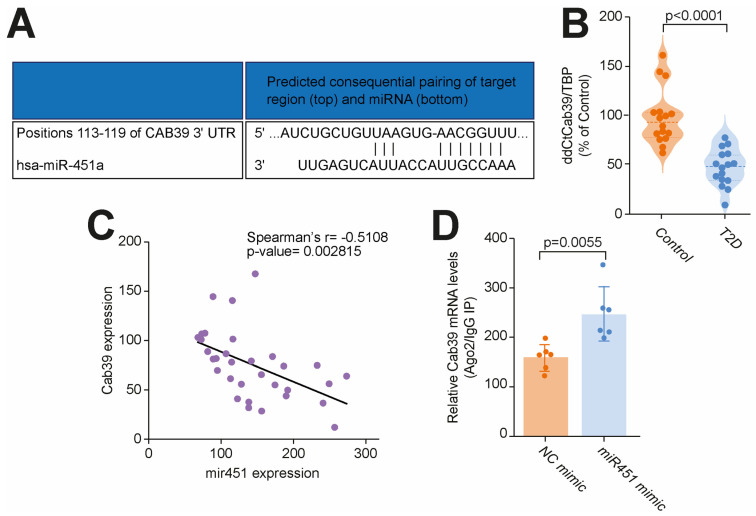
miR-451 orchestrates Cab39 expression in human diabetic heart. (**A**) In silico analysis showing Cab39 to be direct target of miR-451 in humans. (**B**) Real-time PCR showed greater expression of Cab39 in right atrial specimens from T2D patients compared to subjects without diabetes. (**C**) Negative correlation of miR-451 expression with Cab39 levels in myocardial specimens from T2D patients. r indicates Spearman correlation coefficient. (**D**) RISCAgo2 immunoprecipitation, followed by real-time PCR, was employed to measure effects of miR-451 on interaction of Cab39 mRNA with Argonaute 2 in human CMs. Results are presented as mean ± SD and were compared using Student’s *t* test. *p*-value < 0.05 was considered significant. Adjusted *p*-values (alpha/g) are shown.

Calcium-binding protein 39 (Cab39), a scaffold protein of liver kinase B1 (LKB1) involved in the regulation of AMP-activated protein kinase (AMPK) signaling [18], was reported to be a direct target of miR-451 in a mouse model of T2D. In silico analysis confirmed Cab39 to be a direct target of miR-451 in humans (Figure 3A). We next validated the Cab39 expression in our setting and found a significant downregulation of Cab39 in myocardial specimens from the T2D patients vs. the controls without diabetes (Figure 3B). Moreover, we observed a strong negative correlation between the miR-451 levels and Cab39 expression, suggesting a possibility of miR-451-induced Cab39 repression also occurring in humans (Figure 3C). RISC(ago2) immunoprecipitation was employed to demonstrate whether miR-451 affected the interaction of Cab39 mRNA with Argonaute 2 (Ago2), a major component of the RISC complex. The association of Cab39 mRNA with Ago2 was assayed using a real-time PCR of Cab39 mRNA in Ago2 immunoprecipitates (IP). We found that Cab39 mRNA in Ago2 IP samples was enriched by miR-451, thus validating that miR-451 facilitates an endogenous association between Cab39 mRNA and RISC (Figure 3D).

### 3.3. Targeting miR-451 Rescues Diabetes-Related Lipotoxic Damage and Mitochondrial Dysfunction in Human Cardiomyocytes

**Figure 4:**
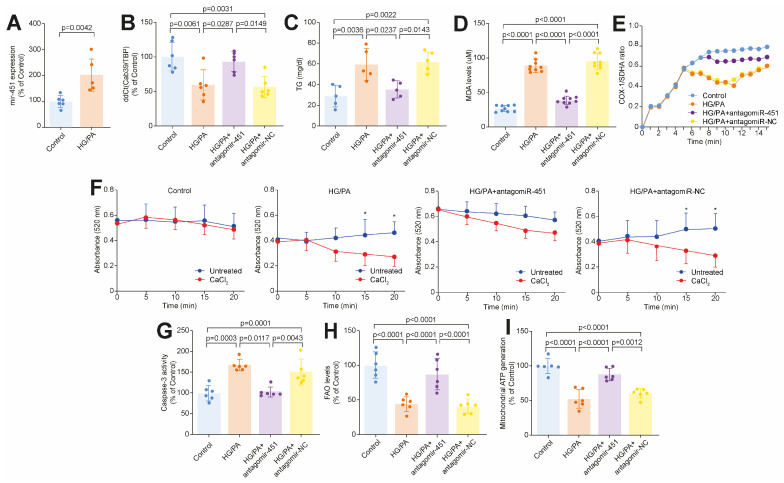
Targeting miR-451 rescues diabetes-induced lipotoxic injury in human cardiomyocytes. (**A**) miR-451 expression in CMs exposed to high glucose concentration and palmitic acid vs. normal glucose concentration (control group). (**B**) Cab39 gene expression in CMs exposed to control conditions or combination of high glucose concentration and palmitic acid, in presence or absence of miR-451 inhibition by specific antagomiR. (**C**) Triglyceride content across 4 experimental groups. (**D**) Lipid peroxidation assessed based on malondialdehyde (MDA) levels in CMs exposed to high glucose concentration and palmitic acid, in presence or absence of miR-451 inhibition. (**E**) miR-451 restores mitochondrial biogenesis in CMs treated with high glucose concentration and palmitic acid. Changes in SDHA and COX-1 levels (MitoBiogenesis assay) were normalized to total cell number. (**F**) Mitochondrial swelling assay. Line graphs represent time-course of decrease in light absorbance with (blue line) and without (red line) calcium overload. * Different from untreated cells at *p* < 0.05. (**G**) CM apoptosis assessed using Caspase-3 assay across 4 experimental groups. (**H**,**I**) Inhibition of miR-451 exerts favorable metabolic effects by restoring fatty acid oxidation and ATP production. Results are presented as mean ± SD and compared using ANOVA followed by Bonferroni post hoc test. *p*-value < 0.05 was considered significant. Adjusted *p*-values (alpha/g) are shown.

To appraise the causal contribution of miR-451 to damage in human cardiomyocytes (CMs), AC16 human cardiomyocytes were cultured in the presence in the presence of high glucose (HG concentration, 25 mM) and palmitic acid (PA, 200 uM) to mimic a T2D-like metabolic environment, characterized by hyperglycemia, fatty acid overload and insulin resistance. In line with our findings in human myocardial samples, miR-451 was upregulated in CMs treated with the combination of a HG concentration and PA compared to those exposed to normal glucose levels (Figure 4A). We next performed miR-451 loss-of-function assays by using an miR-451 antagomiR and found that miR-451 inhibition restored the Cab39 levels (Figure 4B). Moreover, we observed that miR-451 rescues TG accumulation in CMs while blunting oxidative stress, assessed using the MDA levels (Figure 4C,D). Of note, miR-451 inhibition was also associated with an improvement in mitochondrial biogenesis, assessed using the COX1/SDHA ratio, and a significant reduction in the rate of mitochondrial swelling (Figure 4E,F). For the assessment of organelle swelling, isolated mitochondria were challenged with calcium overload, and the rate of swelling was determined usinglight scattering. Organelles from CMs exposed to normal glucose levels (5mM) showed stable absorbance throughout the 20 min time-course. In contrast, HG concentration and PA-treated CMs displayed mitochondrial swelling, which was prevented by miR-451 inhibition (Figure 4F). In line with the preservation of the mitochondrial integrity, an miR-451 blockade also prevented apoptosis in CMs exposed to a HG concentration and PA (Figure 4G). Notably, inhibition of miR-451 also exerted favorable metabolic effects by restoring fatty acid oxidation and ATP production (Figure 4H,I).

**Figure 5:**
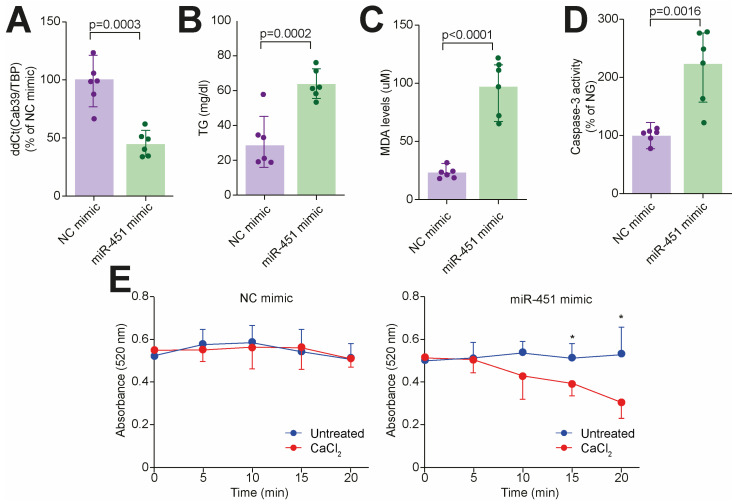
miR-451 mimic recapitulates features of lipotoxic injury in human cardiomyocytes. (**A**) Cab39 gene expression in CMs treated with mir-451 mimic or NC mimic. (**B**) Triglyceride content across 2 experimental groups. (**C**) Lipid peroxidation assessed based on malondialdehyde (MDA) levels in CMs in presence or absence of miR-451 mimic. (**D**) CM apoptosis assessed using Caspase-3 assay across 2 experimental groups. (**E**) Mitochondrial swelling assay. Line graphs represent time-course of decrease in light absorbance with (blue line) and without (red line) calcium overload. * Different from untreated cells at *p* < 0.05. Results are presented as mean ± SD and were compared using Student’s *t* test. *p*-value < 0.05 was considered significant. Adjusted *p*-values (alpha/g) are shown.

**Figure 6:**
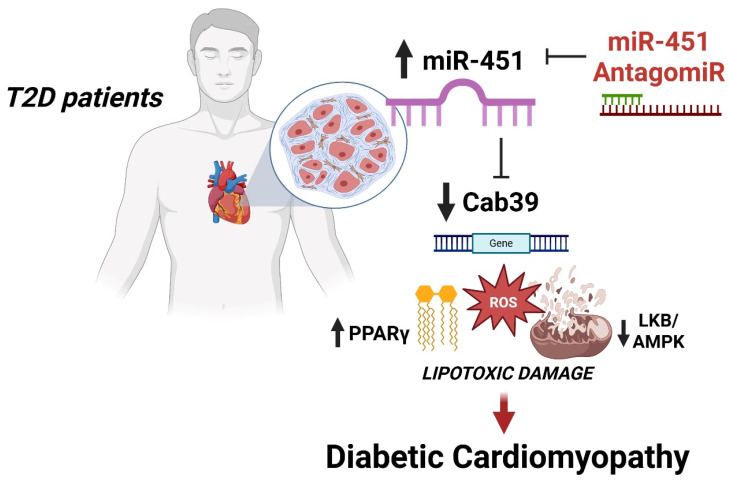
Schematic summarizing the study’s findings. In the human diabetic myocardium, upregulation of miR-451 decreases the levels of the AMPK-activating gene Cab39, thus fostering lipid accumulation, oxidative stress, mitochondrial dysfunction and apoptosis. These events contribute to the development of the diabetic cardiomyopathy phenotype. Targeting miR-451 prevents features of diabetes-related lipotoxic injury.

To further demonstrate the causal role of miR-451 in the induction of lipotoxic injury, we performed gain-of-function assays by using an miR-451 mimic. Of interest, an increase in the endogenous miR-451 levels in cultured human cardiomyocytes led to Cab39 downregulation (Figure 5A) while recapitulating features of diabetes-induced lipotoxic injury, namely TG accumulation, oxidative stress, apoptosis and mitochondrial disruption (Figure 5B–E). Taken together, our findings suggest that miR-451 is an active orchestrator of lipotoxic injury and mitochondrial dysfunction in the human diabetic myocardium (Figure 6).

## 4. Discussion

In the present study we show that miR-451 drives lipotoxic injury in the human diabetic heart. Several lines of evidence support our conclusions: (i) miR-451 was upregulated in myocardial specimens from the patients with T2D compared to the controls without diabetes. (ii) The miR-451 levels positively correlated with cardiometabolic features as well as with TG accumulation and expression of lipid-related transcripts, such as the PPARγ-, PPARα- and PPAR-dependent genes Fas, Cd36, Lpl and Plin5; (iii) miR-451 was found to orchestrate the expression of Cab39, a pivotal regulator of LKB1/AMPK signaling; (iv) targeting miR-451 in human cardiomyocytes prevented major features of lipotoxic damage, including TG accumulation, oxidative stress, mitochondrial dysfunction and apoptosis.

Despite intense research in mouse models of cardiometabolic disease, there remains an overall lack of mechanism-based therapies to be employed in the human setting. Several experimental observations failed to translate into humans, and there is a growing need for new molecular targets to tackle diabetic cardiomyopathy and heart failure in the decades to come. Diabetic cardiomyopathy has been observed in up to 30% of diabetic patients, and its prevalence increases with the duration of diabetes, affecting around 20% of patients after a 10-year disease duration [5,19]. Based on recent projections, 783 million people will have diabetes by the year 2024 and, of these, approximately 230 million will develop features of diabetic cardiomyopathy [1,4]. This specific phenotype is characterized by cardiac metabolic impairment and lipotoxic damage with high levels of oxidative stress and inflammation, as well as left ventricular hypertrophy, diastolic dysfunction and atrial remodeling [4,5]. Patients with diabetic cardiomyopathy are at increased risk of developing heart failure with a preserved ejection fraction (HFpEF), a multisystemic syndrome associated with high morbidity and mortality [20,21,22].

Mounting evidence indicates that exposure to chronic metabolic stress fosters epigenetic changes, ultimately leading to alterations in gene expression and disease phenotypes [23]. Among the different epigenetic signals, non-coding RNAs act as pivotal orchestrators of pathways involved in cardiac damage and are gaining increasing attention [7]. Of clinical relevance, RNA-based therapeutics—which encompass a range of approaches, including small interfering RNA (siRNA), antisense oligonucleotide (ASO) and messenger RNA (mRNA) technologies—have emerged as a promising field with the potential to revolutionize the management of cardiovascular disease [9]. Recent clinical trials have already shown the potential of RNA interference to treat a variety of cardiac phenotypes [13]. Hence, the identification of non-coding RNAs implicated in diabetic cardiomyopathy is of the utmost importance to prevent cardiac dysfunction and HFpEF in people with diabetes.

MicroRNA profiling in heart specimens from mice with diet-induced diabetes revealed miR-451 to be a top-ranking microRNA [12]. In this study, miR-451 was found to drive cardiac dysfunction in mice by inducing metabolic dysfunction and oxidative stress. Interestingly, diabetic mice with cardiomyocyte-specific deletion of miR-451 were protected against the development of diabetic cardiomyopathy, suggesting the causal contribution of this miR to diabetes-induced cardiac damage [12]. This study was one of the most accurate in detecting changes in the microRNA landscape in an experimental model of diet-induced T2D, which reflect features of the human disease, such as obesity, insulin resistance, fatty acid overload and low-grade inflammation. Most studies investigating miRNA changes in diabetic cardiomyopathy have focused on streptozotocin (STZ)-induced diabetes [8,24,25,26], an experimental model which lacks clinical relevance given the presence of STZ-induced toxicity and extremely high blood glucose levels in the absence of obesity and insulin resistance. However, miR-451 was also found to be upregulated in the hearts of mice with STZ-induced diabetes, suggesting that hyperglycemia is an important driver of increases in miR-451 in the diabetic heart [27].

Based on the emerging role of miR-451 in experimental diabetes, in the present study we investigated whether this miR plays a role in the human diabetic myocardium and thus represents a potential therapeutic target in this setting. In heart specimens from patients with T2D, we showed that miR-451 was upregulated and correlated with cardiometabolic features and markers of lipotoxic damage. Mechanistically, we showed that miR-451 targets Cab39, a key regulator of the LKB1/AMPK pathway [12,18]. Of note, targeting miR-451 in human CMs exposed to hyperglycemia and palmitic acid restored the Cab39 levels and prevented TG accumulation and lipotoxic injury as assessed by the levels of oxidative stress, mitochondrial dysfunction and apoptosis. In a study by Kuwabara et al. [12], the ROS levels were higher in high-fat diet (HFD)-fed miR-451 cKO mouse hearts than in control mouse hearts. The authors explained these findings by a possible AMPK-driven increase in mitochondrial function and superoxide production, eventually exerting protective effects. In our study, miR-451 inhibition was associated with improved mitochondrial biogenesis, preserved organelle integrity and reduced ROS, as assessed based on the MDA levels. The different results can be explained by the fact that MDA is a measure of lipid peroxidation and does not specifically reflect the superoxide levels. Moreover, in our model we used a combination of hyperglycemia and palmitic acid, while in the study by Kuwabara et al. [12] only palmitic acid was used. A reduction in lipid peroxidation is beneficial for the heart and can be explained by a reduced fatty acid overload and oxidation within the mitochondria, with lower generation of free radicals and pro-oxidant sphingolipids [2,4].

The LKB1/AMPK pathway is heavily involved in cardiac homeostasis and its activation exerts anti-hypertrophic effects while fostering a favorable metabolic profile in conditions of metabolic stress [28,29]. We showed that miR-451 is a master regulator of Cab39, an LKB1 scaffold protein involved in AMPK activation [18]. Upregulation of miR-451 was found to decrease the Cab39 levels, thus leading to AMPK inhibition. The relevance of our findings is supported by the theory that AMPK deficiency exacerbates obesity-induced cardiac hypertrophy [30]. So far only a few drugs have shown the potential to modulate the AMPK activity in experimental models. Metformin, a widely used antidiabetic drug, has been shown to activate AMPK signaling in cardiomyocytes, a phenomenon which may contribute to explaining the beneficial effects of this drug on heart failure outcomes, as outlined in recent meta-analyses [31]. However, metformin is not a specific activator of AMPK. Our results provide new insights regarding the potential to modulate the miR-451 levels in T2D patients to boost AMPK signaling in the heart, thus leading to reduced lipotoxic damage and improved cardiac remodeling.

Our study is the first to assess the miR-451 levels in the human heart and, more specifically, in the setting of diabetic cardiomyopathy. The strength and novelty of our work is demonstrated by our successful validation of the microRNA-451 (miRNA-451) levels in human hearts, which has revealed a new molecular target for preventing diabetic cardiomyopathy. Although our findings need to be confirmed by other studies, these results encourage efforts to better characterize miR-451 as a driver of metabolic cardiomyopathy and HFpEF. An important outcome of this study was the identification of miR-451’s potential for use as a circulating biomarker of cardiac lipotoxic injury. Previous work has shown that miR-451 is detectable in human plasma and is associated with several disease states, including inflammatory and rheumatic diseases, viral infections and cancer [32,33]. Hence, our results set the stage for the development of new personalized epigenetic biomarkers and therapeutic targets to diagnose and treat cardiac disease in people with type 2 diabetes.

Our work has some limitations. First, miR-451 upregulation was not determined in an unbiased way. Our approach did not allow us to appreciate the differential contribution of other miRs to the observed phenotype. However, miRNA profiling in T2D mouse hearts has revealed miR-451 to be one of the top-ranking upregulated miRNAs. Future work is needed to unveil the miRNA landscape of the diabetic human heart. This approach will be invaluable for the identification of other miRNA signatures potentially involved in diabetes-related cardiac remodeling and dysfunction. Moreover, it will allow us to understand the relative expression of miR-451 in comparison with that of other miRNA signatures. Second, miR-451 was detected in the atrial myocardium, a tissue which shares some but not all the features of the left ventricular myocardium. However, obtaining left ventricular myocardial specimens from T2D patients is quite difficult and this poses further challenges for the recruitment of age-matched controls without diabetes. Based on this limitation, we cannot conclude that miR-451 is directly involved in human ventricular remodeling and dysfunction, and further work is needed in this direction. Previous experimental work has shown that miR-451 is upregulated in left ventricular specimens from diabetic mice, suggesting a role for this miR in the ventricular myocardium. On the other hand, our findings provide novel insights into the implications of lipotoxic injury and CM dysfunction in the atria and how these molecular alterations contribute to the increased risk of atrial fibrillation in diabetic patients. Although Cab39 has been reported to act as a key downstream effector of miR-451, in our study we did not perform rescuing experiments demonstrating the causal contribution of this protein to rescuing diabetes-induced lipotoxic injury. Finally, our observations were focused on post-transcriptional regulation of gene expression by miR-451, and protein expression data were not provided. However, previous work has shown a strong concordance between the gene and protein expression levels of Cab39 and AMPK in experimental models of diabetes [12].

In conclusion, our study sheds light on a new mechanism driving diabetic cardiomyopathy in humans and sets the stage for larger preclinical investigations testing this intervention for the prevention of diabetic cardiomyopathy and heart failure in T2D patients.

## Figures and Tables

**Table 1:** Demographics, laboratory parameters, medications and echocardiographic data for patients with and without diabetes. Values are expressed as mean ± SD. *p*-values refer to those obtained using Student’s *t* and Chi2 tests. BMI, body mass index; FPG, fasting plasma glucose; LDL-C, low-density lipoprotein cholesterol; HDL-C, high-density lipoprotein cholesterol; ACE-is, angiotensin-converting enzyme inhibitors; ARBs, angiotensin receptor blockers; GLP1RA, glucagon-like peptide-1 receptor agonist.

	Controls (n = 16)	T2D (n = 16)	*p*-Value
Demographics			
Age (years)	59.8 ± 8.1	61.1 ± 9.1	0.65
Gender (F:M)	10:5	13:4	0.54
BMI (kg/m^2^)	28.5 ± 3.4	30.1 ± 6.2	0.38
Systolic blood pressure (mmHg)	122 ± 10	119 ± 31	0.68
Diastolic blood pressure (mmHg)	72.3 ± 10.2	73.1 ± 7.9	0.8
Laboratory parameters			
FPG (mg/dL)	93 ± 11	157 ± 40	<0.01
HbA1c (%)	5.6 ± 0.37	7.0 ± 0.82	<0.01
Duration of diabetes (years)	0.0 ± 0.0	5.8 ± 4.1	NA
Triglycerides (mg/dL)	87 ± 31	138 ± 52	0.002
Total cholesterol (mg/dL)	177 ± 33	169. ± 45	0.54
Medications			
Statins (%)	31.3	43.8	NS
ACE-is/ARBs (%)	50.0	62.5	NS
Beta-blockers (%)	50.0	68.8	NS
Diuretics (%)	25.0	37.5	NS
Metformin (%)	12.5	37.5	<0.01
Insulin (%)	0.0	43.8	<0.01
GLP1RA (%)	0.0	6.25	NS

## Data Availability

The original contributions presented in this study are included in the article/supplementary material. Further inquiries can be directed to the corresponding author.

## Supplementary Materials

The following supporting information can be downloaded at: https://www.mdpi.com/article/10.3390/cells14171401/s1, Table S1. Human primers used for real time PCR.

## Abbreviations

miR	microRNA
T2D	type 2 diabetes
HF	heart failure
HFpEF	heart failure with a preserved ejection fraction
HG	high glucose
NG	normal glucose
PA	palmitic acid
CMs	cardiomyocytes
AMPK	adenosine monophosphate-activated protein kinase
LKB1	liver kinase B1
Cab39	calcium-binding protein 39
TG	triglyceride
HFD	high-fat diet
CABG	coronary artery bypass grafting
