# Supplementary material for: miR-451 Is a Driver of Lipotoxic Injury in Patients with Diabetic Cardiomyopathy
Source: Cells. 2025 Sep 8;14(17):1401. doi: 10.3390/cells14171401 (PMC12427974; doi:10.3390/cells14171401)
Supplement: Supplementary file 1 [file cells-14-01401-s001.zip › cells-3803023-supplementary.pdf]

SUPPLEMENTAL MATERIAL

Supplemental Table

Ppara Forward	5' – AAA CTG ACA TTG GGC ACG TC – 3'
Ppara Reverse	5' – CTG TTT CCA TCC GCA TCT GG – 3'
Pparg Forward	5' – ATG CAG TGG TCT CAG TAC CC – 3'
Ppargc Reverse	5' – TTG GTG TGA GGA GGG TCA TC – 3'
Cab39 Forward	5' – ATG AAC CTG CTG CGA GAC AA – 3'
Cab39 Reverse	5' – AAA CTG CTCA TCC TCC GTC C – 3'
Cd36 Forward	5' – CTC AAT GTC GTC GCT TCT GG – 3'
Cd36 Reverse	5' – AAT GCAGCT ACC TTT GAG CC– 3'
Fas Forward	5' – GCC TTT GAA ATG TGC TCC CA – 3'
Fas Reverse	5' – GTG AAC TGC TGC ACG AAG AA– 3'
Plin5 Forward	5' – CTG TTT GCA GCA CGA TGT CT – 3'
Plin5 Reverse	5' – CTG TCC TTG GCT GCA CTG TA – 3'
Lpl Forward	5' – AGT CCC AGC TTT GTC ATC GA – 3'
Lpl Reverse	5' – TCG AAG TTC TGG CAC ATC AG – 3'
GAPDH Forward	5' – AGG GCT GCT TTT AAC TCT GGT – 3'
GAPDH Reverse	5' – CCC CAC TTG ATT TTG GAG GGA – 3'

Table S1. Human primers used for real time PCR.
